# Correction: Alhamhoom et al. Synergistic Antihyperglycemic and Antihyperlipidemic Effect of Polyherbal and Allopolyherbal Formulation. *Pharmaceuticals* 2023, *16*, 1368

**DOI:** 10.3390/ph19081262

**Published:** 2026-08-11

**Authors:** Yahya Alhamhoom, Syed Sagheer Ahmed, Rupesh Kumar M., MD. Salahuddin, Bharathi D. R., Mohammed Muqtader Ahmed, Syeda Ayesha Farhana, Mohamed Rahamathulla

**Affiliations:** 1 Department of Pharmaceutics, College of Pharmacy, King Khalid University, Al Faraa, Abha 62223, Saudi Arabia; ysalhamhoom@kku.edu.sa; 2 Department of Pharmacology, Sri Adichunchanagiri College of Pharmacy, Adichunchanagiri University, BG Nagara, Mandya 571448, India; rambha.eesh@gmail.com; 3 Department of Pharmacology, Alameen College of Pharmacy, Bengalore 560027, India; manirupeshkumar@yahoo.in; 4 Department of Chemistry, Alameen College of Pharmacy, Bengalore 560027, India; rcsalahuddin@alameenpharmacy.edu; 5 Department of Pharmaceutics, College of Pharmacy, Prince Sattam Bin Abdul Aziz University, Al Kharj 11942, Saudi Arabia; muqtadernano@psau.edu.sa; 6 Department of Pharmaceutics, Unaizah College of Pharmacy, Qassim University, Unaizah 51911, Saudi Arabia; a.farhana@qu.edu.sa

**References**

In the published manuscript [1], reference 36 has been retracted and should be deleted:

36. Prabhu, S.; Vinodhini, S.; Elanchezhiyan, C.; Rajeswari, D. Evaluation of antidiabetic activity of biologically synthesized silver nanoparticles using Pouteria sapota in streptozotocin-induced diabetic rats. *J. Diabetes* **2018**, *10*, 28–42.

With this correction, the order of some references has been adjusted accordingly. The authors state that the scientific conclusions are unaffected. This correction was approved by the Academic Editor. The original publication has also been updated.
